# Establishment of a tumor-associated fibroblast associated gene score based on scRNA-seq to predict prognosis in patients with triple-negative breast cancer

**DOI:** 10.1371/journal.pone.0311801

**Published:** 2024-10-17

**Authors:** Hao Yu, Ziqi Peng, Xing Li, Yiqi Zhang

**Affiliations:** 1 Department of Breast Surgery, The First Affiliated Hospital of Jinzhou Medical University, Jinzhou, China; 2 Department of Breast Surgery, The First Affiliated Hospital of China Medical University, Shenyang, China; Kyoto Prefectural University of Medicine, JAPAN

## Abstract

The tumor microenvironment (TME) is emerging as a tool for the development of improved patient prognosis and the development of novel antitumor drugs. As the most important stromal cells in the tumor microenvironment, cancer-associated fibroblasts (CAFs) play an important role in the development of TNBC. The rise of single-cell sequencing technology has facilitated our study of the various cell types in TME. In this study, we interpreted the heterogeneity of TNBCs from the perspective of tumor-associated fibroblasts in the tumor microenvironment based on the TNBC single-cell sequencing dataset GSE118389, in the hope of providing help for individualised treatment. Combining the TCGA database and the GSE103091 dataset, four genes associated with CAFs in TNBC (CERCAM, KLF10, ECM1,HGF) were identified using the R package Seurat as well as correlation consensus clustering analysis. Meanwhile, qRT-PCR, WB and IHC experiments confirmed their expression in TNBC. Based on these genes, CAFs Score was established and validated to correlate with the prognosis of patients with TNBC, with patients in the high score group surviving significantly worse than those in the low score group (P<0.001). In addition, there were significant differences in immune cell infiltration and expression of immune checkpoints between the high and low scoring groups. Compared to Stage I & II, the CAFs Score was higher in Stage III & IV TNBC patients (P = 0.043) and higher in N1-3 TNBC patients than in N0 TNBC patients (P = 0.035). EMT scores were higher within the high CAFs Score group (P = 1.4e-11) and there was a positive correlation between Stemness Score and CAFs Score (R = 0.61, P = 3.6e-09). Drug sensitivity analysis combining the GSE128099 showed a higher sensitivity to Gemcitabine in the low CAFs Score group (P = 0.0048). We speculate that these four CAFs-related genes are likely to be involved in regulating gemcitabine resistance in TNBC patients.

## 1 Introduction

Breast cancer has overtaken lung cancer to become the world’s largest cancer, according to global cancer data for 2020 [1]. Triple negative breast cancer (TNBC) is a molecular subtype with high invasion and metastasis, poor prognosis and lack of specific therapeutic targets [2],has always been a hot spot for people to explore actively. In recent years, with the development of precision medicine, people try to divide TNBC into different subtypes at different levels in the hope of individualized treatment. Typically, in 2011, the US team classified TNBC into seven distinct subtypes [3], according to the gene expression profiling; in 2019, the Chinese Fudan cancer team classified TNBC into four different types [4] including luminal androgen receptor (LAR).The researchers have continuously analyzed the heterogeneity of TNBC from all levels, hoping to be helpful to clinical research, improve treatment effect and improve the prognosis of patients with TNBC.

Tumor microenvironment (TME) plays an important role in the process of tumorigenesis and development. The microenvironment includes not only the internal environment of the tumor cells, but also the external environment composed of the surrounding stromal cells, extracellular matrix (ECM) and so on. Tumor cells and their internal and external environments together form a functional whole and interact with each other to promote tumor progression. With the increasing understanding of TME in recent years, TME has gradually become a tool for developing new anti-tumor drugs and improving patients’ prognosis [5]. As the most important stromal cell in TME, tumor-associated fibroblasts (CAFs) have been shown to play an important role in promoting the progression of breast cancer. In addition, the presence of CAFs in the matrix of breast cancer is associated with poor prognosis [6, 7]. CAFs has a variety of carcinogenesis mechanisms, it can enhance the synthesis and remodeling of breast cancer extracellular matrix through a variety of ways to promote the progressive growth of tumors [8]; It can enhance the adhesion of endothelial cells and the invasion of cancer cells by changing the immune cell environment to the tumor-tolerant phenotype [6]; It can also promote epithelial-mesenchymal transformation (EMT) and phenotype transformation of tumor stem cells to promote tumor metastasis [9].

In recent years, the rise of single-cell sequencing technology has made it possible to extract and amplify genomes or transcriptomes at the single-cell level and to perform high-throughput sequencing [10],it is also convenient for us to study various kinds of cells in TME. Previous studies on the heterogeneity of TNBC are still limited to the tumor cells themselves, and TME has a strong potential therapeutic value, especially CAFs.

## 2 Materials and methods

### 2.1 Data collection

Download single cell sequencing (scRNA-Seq) data for TNBC patients with the number GSE118389 (https://www.ncbi.nlm.nih.gov/geo/query/acc.cgi?acc=GSE118389) from the GEO Database (https://www.ncbi.nlm.nih.gov/geo/). The data were preprocessed with the R package "Limma", and the expression data of the same gene name were averaged. Download the transcriptome data (RNA-seq; Fragments Per Kilobase Million [FPKM] value) and clinical information for breast cancer patients from the TCGA database (https://portal.gdc.cancer.gov/). Only patient data with complete clinical information is retained. Combined with the pathological information of the patients, the expression data of TNBC were screened.

In addition, RNA-seq and related clinical data of TNBC patients with the number GSE103091 (https://www.ncbi.nlm.nih.gov/geo/query/acc.cgi?acc=GSE103091) and GSE128099 (https://www.ncbi.nlm.nih.gov/geo/query/acc.cgi?acc=GSE128099) in GEO database were selected as the validation set. All data are fully available without restriction.

### 2.2 Identification of TNBC cell subtypes from scRNA-seq data

The scRNA-Seq data was analyzed using the RSEURAT package, and samples with mitochondrial gene percentages greater than 5 were excluded. Use the ‘NORMALIZEDATA’ function to standardize the data and extract 1500 genes with a high coefficient of variation between cells. Then PCA was used to analyze the data and pregroup cells, and the P value of each principal component was obtained. The principal component with a P value less than 0.05 was selected for the next tSNE analysis and the cells were divided into different clusters. Analysis different expressed genes between different clusters, the genes with p less than 0.05 and log2 | FC | greater than 1 are the maker gene of the cluster. The cell types of the cluster are then annotated using R-package ‘singleR’ (SingleR is an R package for automated cell type annotation of scRNA-seq data. Using cell samples with known type labels as reference data sets, the test data set’s cells were labeled and annotated accordingly.) to obtain the different cell components, and the differential genes between the different cell components are analyzed, and we end up with marker genes in different kinds of cells. The genes specifically expressed by different kinds of cells compared with other cells are considered as marker genes.

### 2.3 Screening of key genes using TCGA-TNBC RNA-seq data

According to the gene expression data of TNBC patients in TCGA database (TCGA-TNBC), and the gene expression data of normal breast tissue in TCGA, we analyzed the differential expression of maker gene in CAFs (P<0.05, log2|FC|>1). At the same time, Uni-Cox analysis was used to analyze the genes in maker genes that were associated with the prognosis of patients. These results were cross-linked and genes with inconsistent expression levels and prognostic effects were removed. The FPKM data in TCGA database was transformed into TPM format, and the ConsensusClusterPlus package was used to cluster the genes in the intersection, and the patients with TCGA-TNBC were divided into different clusters. Using K-M survival analysis to judge the difference of survival time among different clusters. Then 1000 Lasso analyses were performed to select the most stable gene hub as gene hub. Finally, the final hub genes were analyzed by consistent cluster analysis again to determine the survival differences among clusters.

### 2.4 Verification of grouping accuracy based on hub genes

The differentially expressed genes between clusterA and clusterB was analyzed by using the R packet ‘limma’ (p<0.05, log2|FC|>1), enrichment analysis (GO and KEGG) of differential genes using the R packet "Clusterprofiler". Wilson Cox was used to compare the expression differences of CAFs marker genes between clusterA and clusterB.EPIC was used to score the TME of TCGA-TNBC patients, and the CAFs related score was extracted. The difference between clusterA and clusterB was analyzed based on the patients’ grouping information. Then, to verify the reliability of the data set, we use the TNBC data of GEO database with the number GSE103091 as the verification set to verify the grouping based on hub genes and the correlation of prognosis.

### 2.5 Generation and correlation analysis of CAFs Score in patients with TNBC

Based on the previous lasso cox analysis, we got the coefficients for each gene, and then we constructed the CAFs Score based on the coefficients and their corresponding gene expression values. The calculation formula is as follows:

CAFsScore=∑iCoefficient(mRNAi)×Ecpression(mRNAi)

In combination with patients’ survival, we used the ‘surv_cutpoint’ function in R package ‘survival’ to select the best cut off value and then divided patients into high and low score groups and analyzed the differences in patient survival between the two groups.

To verify the predictive ability of our constructed CAFs scores, PCA analysis was used, verify that TCGA-TNBC patients can be divided into two clusters based on four hub genes and that the CAFs Score is different between the two clusters. Then, to verify the reliability of our score for survival, ROC analysis was performed and the AUC value of CAFs Score at 1.3.5 years was obtained in patients with TCGA-TNBC (Generally, a value greater than 0.75 is considered credible).

### 2.6 Relationship between CAFs Score and immunity

A total of 39 kinds of immune cells and stromal cells in tumor microenvironment of patients with TCGA-TNBC were evaluated by using R packet ‘xCELL’ (xCELL is an immune cell infiltration assessment tool based on gene expression data that identifies potential immune cell subpopulations and calculates their relative abundance in tissues), wilson cox was used to examine the differences in the scores of immune cells and stromal cells in patients with high and low CAFs scores. At the same time, the expression data of immune checkpoint gene were extracted and the difference of immune checkpoint level between high and low score groups was calculated, P<0.05 was considered to have statistical significance.

### 2.7 Relationship between CAFS Score and EMT, characteristics of tumor stem cells

The clinicopathologic information of TCGA-TNBC patients was extracted and the differences of CAFs Score among TNBC patients with different grades and TNM stages were compared.

Subsequently, marker genes of the pathway named ‘HALLMARK_EPITHELIAL_MESENCHYMAL_TRANSITION’ were obtained from the GSEA database (http://www.gsea-msigdb.org/), and the EMT score of TNBC patients was obtained by ssGSEA analysis,the difference of EMT score between low CAFs Score group and high CAFs Score group was analyzed by Wilson cox, P<0.05 was considered significant. The stem cell group and its differentiated ectoderm, mesoderm and endoderm progenitor cell information in PCBC database were used as the initial data set, and the driness index was derived using OCLR algorithm training. Then, the transcriptome expression corresponding to the driness index calculated based on OCLR was applied to the TCGA dataset to calculate the mRNAsi of each sample.

The results showed that there was heterogeneity in breast cancer stem cells [11], with high expression of Vimentin (PRELID1) in mesenchymal breast cancer stem cells, high expression of E-cadherin (CDH1) and ALDHA1 in epithelial breast cancer stem cells [12].The expression data of these three genes in patients with TNBC were extracted, and the expression differences of these three genes between high and low CAFs Score groups were calculated, P<0.05 was considered to be statistically significant.

### 2.8 Determination of the sensitivity of patients in high and low groups to tumor treatment drugs

PRRophetic is an R package created from gene expression and drug sensitivity data of cell lines from the Cancer Genome Project (CGP) to predict clinical chemotherapeutic responses from tumor gene expression levels [13].The sensitivity of patients with TNBC in high and low score groups to CAFs-related chemotherapeutic drugs was analyzed with this package (P<0.05 was considered significant).

### 2.9 Quantitative reverse transcription polymerase chain reaction (qRT-PCR)

Total RNA was extracted by TRIzol (Invitrogen) and reverse transcripted according to the instruction manual of Toyobo. The expression of mRNA was detected using SYBR (Toyobo) and was calculated using 2^-ΔΔCT^ method. The primers used were as follows: CERCAM: forward, GAGCCCAGGTTCTACCCAGAT; reverse, GCAGAGTCTGATTGTTGGTCA. ECM1: forward, GATATTCCCGCTGCTGCCACTG; reverse, TCACAGAATCGGCTCATTGCTTCC. HGF: forward, TCCAAGGTCAAGGAGAAGGCTACAG; reverse, CAGGAGTCATGTCATGCTCGTGAG. KLF10: forward, GCAGCCAGCATCCTCAACTATCAG; reverse, TGACACAGCGGCACATGGTATG. β-actin: forward, CTGGCCGGGACCTGACT; reverse, TCCTTAATGTCACGCACGATTT.

### 2.10 Western blot

All proteins were extracted using RIPA lysis buffer (Beyotime) and quantified by BCA protein quantification method. Denaturated proteins were transferred to polyvinylidene fluoride (PVDF) membranes (Millipore) after being separated by sodium dodecyl sulphate–polyacrylamide gel (SDS-PAGE) electrophoresis. After incubated with primary antibodies overnight, the PVDF membrane was incubated with secondary antibodies at room temperature. The signals were further examined using an enhanced chemiluminescence system (Tanon). The antibodies using were as follows: CERCAM (16411-1-AP, Proteintech), ECM1 (11521-1-AP, Proteintech), HGF (ab178395, Abcam), KLF10 (29709-1-AP, Proteintech) and β-actin (66009-1-Ig, Proteintech). The study was approved by the Ethics Committee of the First Affiliated Hospital of Jinzhou Medical University.

### 2.11 Immunohistochemical staining (IHC)

IHC was performed using Ultra-sensitivet S-P Kit (Maixin-Bio, China). Briefly, sections from paraffin embedded TNBC tissues were incubated with primary antibodies against CERCAM (HPA021657, Sigma), ECM1 (SAB2109063, Sigma), HGF (HPA044088, Sigma), KLF10 (2 SAB2106445, Sigma) overnight at 4°C. The images were taken by Olympus microscope.

## 3 Results

### 3.1 Data downloading and collection

**Fig 1:**
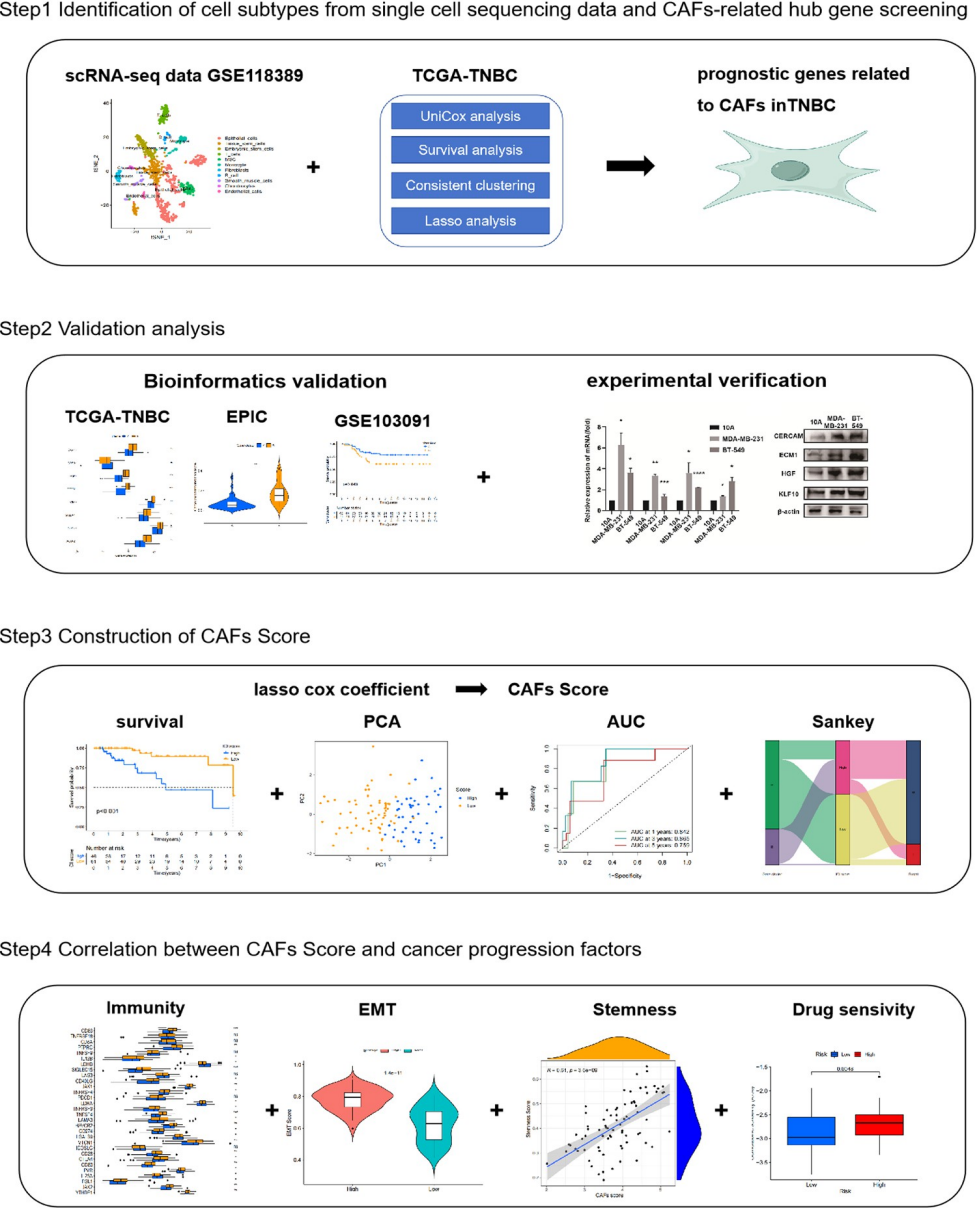
The flowchart of this study.

The scRNA-Seq data from the dataset GSE118389 included 1,534 cells from 6 TNBC patients. Bulk RNA-Seq came from the TCGA and GEO databases, respectively, which contained 116 TNBC samples and 113 adjacent normal tissue samples.The GSE103091 dataset contains gene expression data and clinical information from 107 TNBC patients. Patients in TCGA and GEO databases are listed in Table 1. In this study, mRNA expression before and after gemcitabine treatment was extracted from the NC group in GSE128099, and the differences were analyzed. The flow chart of this study is shown in Fig 1.

**Table 1:** Clinical characteristics of the TNBC patients used in this study.

	TCGA	GSE103091
**Survival status**		
Alive	98	78
Dead	18	29
**Age(median, range)**	53.5(29–90)	57(28–85)
**Stage**		
I	19	NA
II	73	NA
III	19	NA
IV	2	NA
Uknown	3	NA
**T**		
1	26	NA
2	74	NA
3	12	NA
4	4	NA
**N**		
0	74	NA
1	26	NA
2	12	NA
3	4	NA
**M**		
0	99	NA
1	2	NA
MX	15	NA

### 3.2 Identification of different cell types in triple negative breast cancer

**Fig 2:**
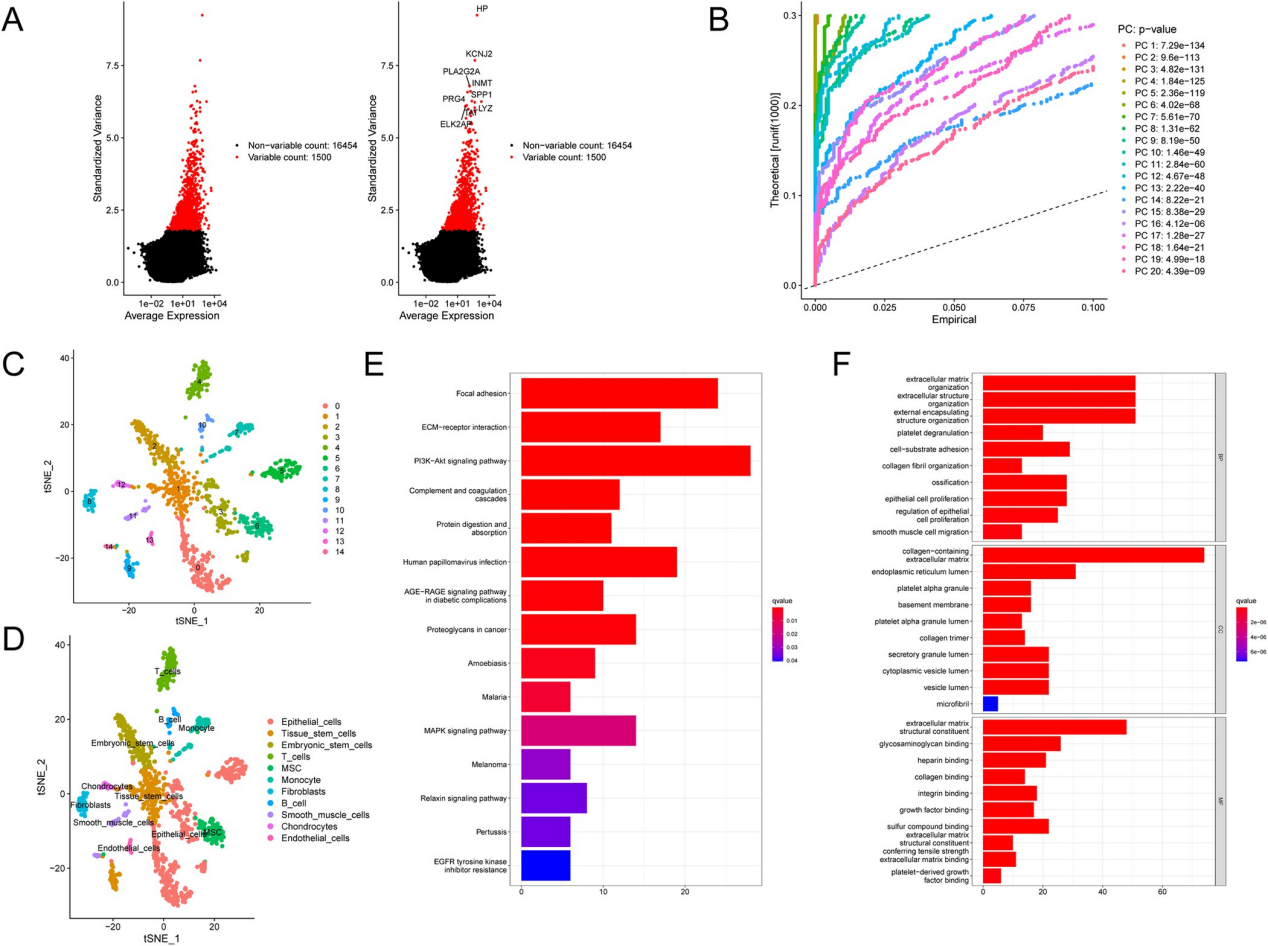
Classification of triple-negative breast cancer tissue cells using single-cell sequencing data. (A) Distribution of 1500 genes with large intercellular coefficients of variation. (B) Principal component analysis of all cells and P values of each principal component. (C) The TSNE algorithm divides the cells into 15 clusters. (D) Cell types in different clusters. (E) KEGG analysis results of marker genes of CAFs cluster. (F) GO analysis results of marker genes of CAFs cluster.

The R package ‘Seurat’ was used to analyze single cell sequencing data for TNBC, and the ‘FindVariableFeatures’ function was used to extract the top 1500 genes with the highest coefficient of variation. The distribution of the 1500 genes is shown in Fig 2A. The P values of the first 20 principal components were analyzed by PCA according to the selected genes (Fig 2B). The 20 principal components were then incorporated into tSNE analysis to calculate proximity, modularize and group the cells. The results showed that 1,534 cells were divided into 15 clusters (cluster 0–14, Fig 2C). Then, the differentially expressed genes (P < 0.05) in each cluster were analyzed and defined as marker genes in each cluster,information of the hub genes of the different clusters are provided in S1 Table. To explore the properties of cells within each cluster, we annotate cell types using the R packet ‘SingleR’. The results show that 15 clusters are defined as 11 cell types, with cluster0 as epithelial cells, cluster1, and cluster9 as tissue stem cells, cluster 3,5 are epithelial cells, cluster 4 is T cell, cluster 8 is fibroblasts, and so on (Fig 2D).Since CAFs is the main component of TME and can promote tumor progression, we chose CAFs module, marker genes in cluster8 for function enrichment analysis, the results shown that these genes are enriched in multiple pathways and biological processes associated with CAFs (Fig 2E and 2F).

### 3.3 Identification of CAFs subtypes in TCGA-TNBC

**Fig 3:**
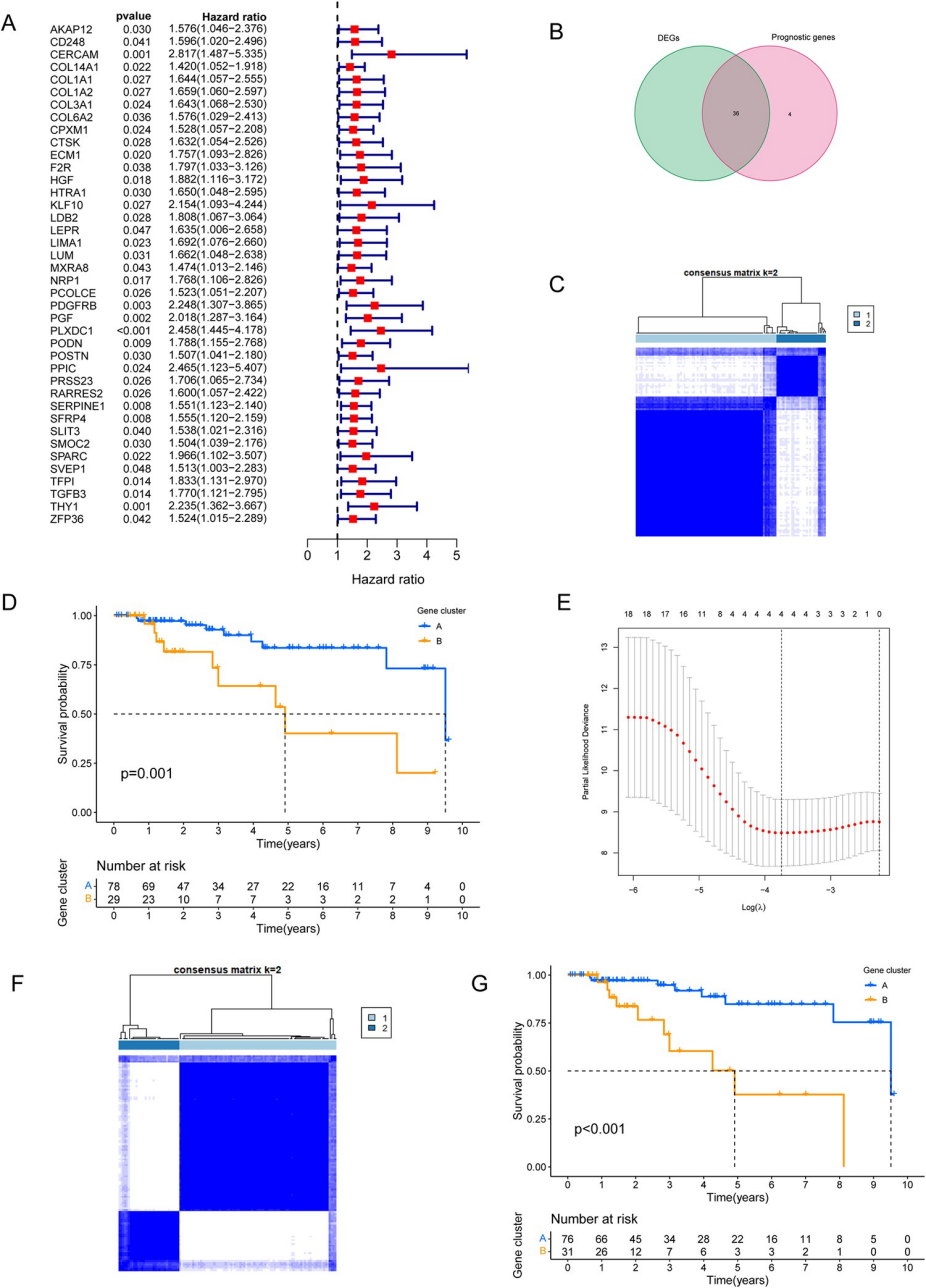
Screening and subtype construction of CAFS-related genes. (A) Univariate COX screening of CAFs marker genes associated with patient survival. (B) Intersection of prognostic marker genes and differentially expressed marker genes. (C,D) Classification of TCGA-TNBC based on 36 intersection genes and survival differences of patients within different types. (E)The most stable CAFS-related genes were screened by lasso analysis. (F,G) Classification of TCGA-TNBC based on the four most stable CAFS-related genes and survival differences among patients within different types.

Expression data of marker genes were extracted from TCGA-TNBC, and univariate cox regression analysis was performed based on patients’ total survival time, a total of 40 overall survival (OS) related genes were identified (Fig 3A). The difference gene and the prognosis-related gene were cross-linked. At the same time, if the gene is overexpressed in the tumor, but the UniCox analysis is a prognostic factor, the gene will be deleted. Similarly, genes with low expression in tumors that Uniox analyzed as prognostic risk factors will be deleted as well, resulting in a total of 36 overlapping genes (Fig 3B). Based on the expression of the above 36 genes, we performed consistent clustering based on the k value. According to the cumulative distribution function, we selected k = 2 as the optimal parameter and divided TCGA-TNBC patients into two groups named clusterA and cluster (Fig 3C).

Furthermore, survival analysis showed that there was significant difference in the OS between clusterA and clusterB patients (P = 0.001), as shown in Fig 3D. We conclude that these 36 genes may affect OS in patients with TNBC through a CAFs-related pathway. Further, through 1000 lasso analyses, four hub genes were screened out (Fig 3E): CERCAM, KLF10, ECM1,HGF. They are the most stable prognostic genes associated with CAFs in TNBC.

In order to explore the value of these four genes, according to the expression of these four genes, we applied consistent cluster analysis based on k value, selected k = 2 as the optimal parameter, and divided TCGA-TNBC patients into two group called clusterA and cluster (Fig 3F).The results of survival analysis showed that the OS of patients in the clusterB was significantly lower than that in clusterA, the difference was statistically significant (P < 0.001, Fig 3G).

### 3.4 Validation of selected hub genes

**Fig 4:**
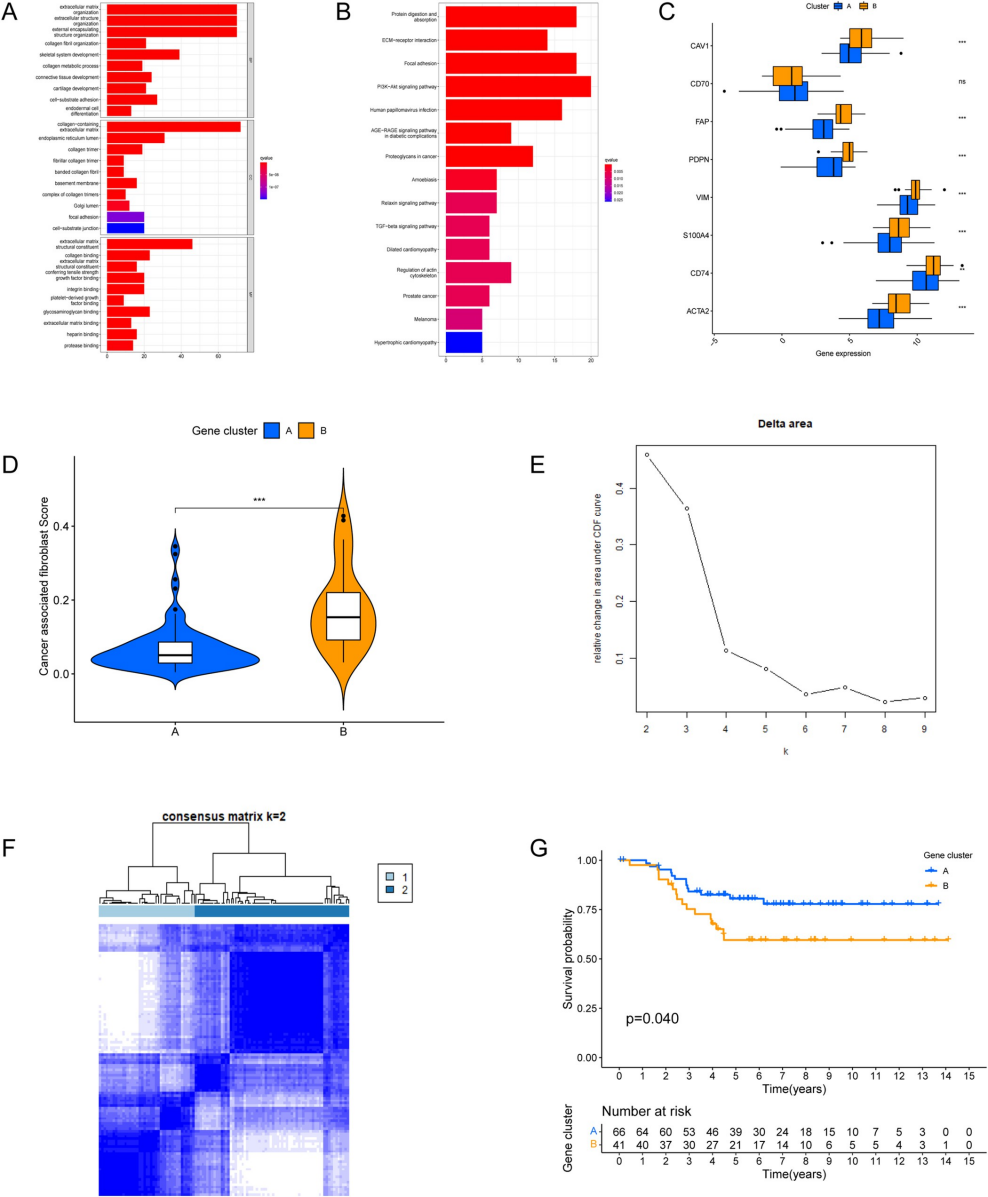
Validation of TCGA-TNBC typing results based on the four most stable CAFS-related genes. (A,B)GO and KEGG analysis results of differential genes among different clusters of TCGA patients. (C) Difference analysis of CAFs marker genes among different TCGA-TNBC clusters. (D) Differences in CAFs scores among different TCGA-TNBC clusters. (E, F, G) Use GEO database data to verify TCGA typing results (E: K-value distribution of GEO patients, F: Classification of GEO patients at k = 2, G: Survival analysis of patients in different clusters).

**Fig 5:**
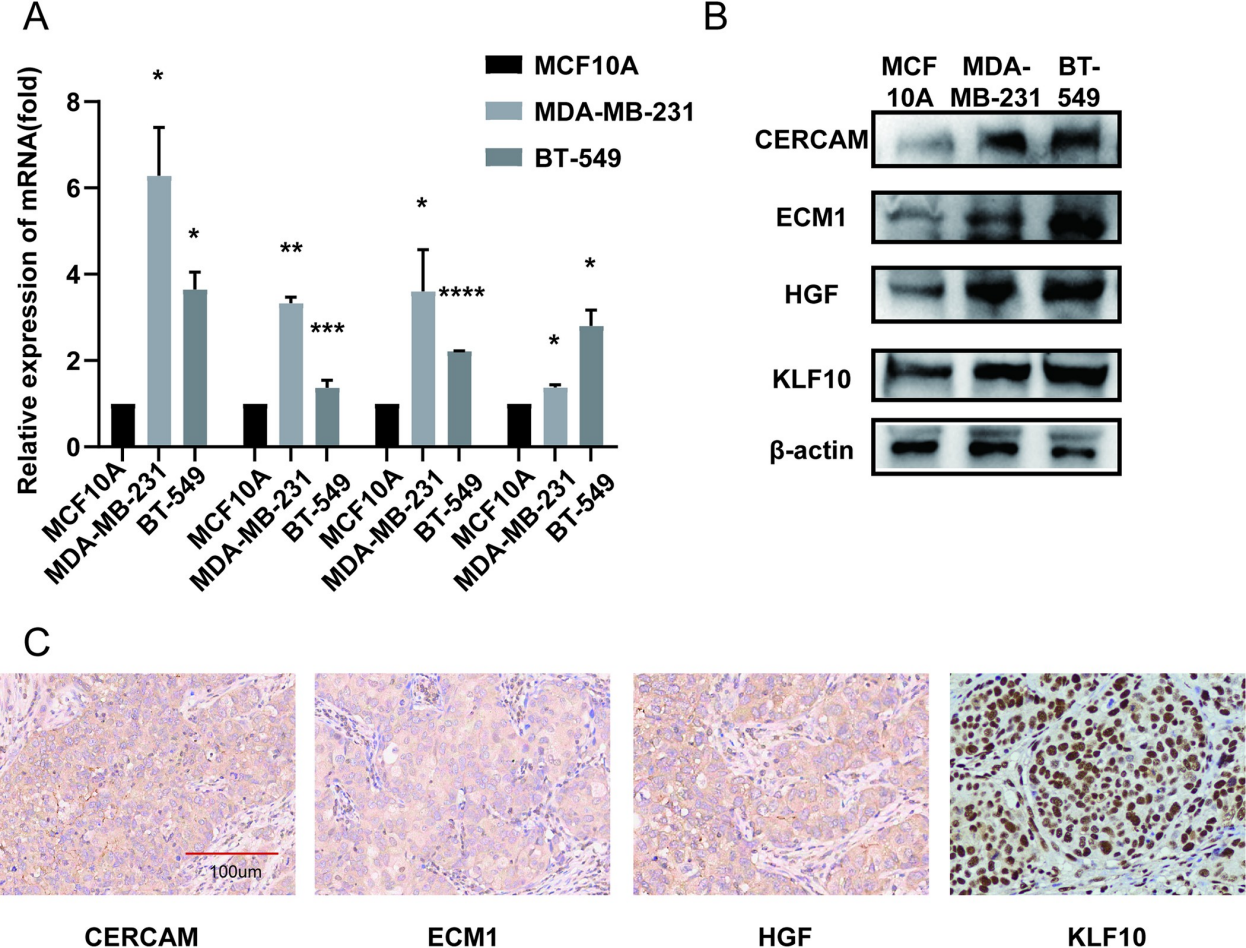
The expression of CERCAM, ECM1, HGF and KLF10 in breast cancer cells. (A) The mRNA expression of CERCAM, ECM1, HGF and KLF10 are higher in breast cancer cells. (B) The protein expression of CERCAM, ECM1, HGF and KLF10 are higher in breast cancer cells. (C) The protein CERCAM, ECM1, HGF and KLF10 protein are positively expressed in TNBC tissues.

The results showed that there were 223 differentially expressed genes (S2 Table) between clusterA and clusterB. The results of enrichment analysis showed that these differentially expressed genes were mainly enriched in CAFs-related pathways and biological processes such as ECM receptor interaction and focal adhesion (Fig 4A and 4B). The expression level of CAFs-related marker genes between clusterA and clusterB was analyzed. The results showed that 7 of 8 marker genes were highly expressed in cluster (P<0.05, Fig 4C). The tumor microenvironment of clusterA and clusterB were rated by Epic website respectively. The results showed that CAFs Score in Epic was higher in clusterB, the difference was statistically significant (P<0.001, Fig 4D). In order to prove the accuracy of the data analysis in the TCGA database, we select the TNBC data of GEO Database GSE103091 to prove that the change of the dataset does not affect our conclusion. In GSE103091, we chose k = 2 as the optimal parameter, according to the cumulative distribution function (CDF) (Fig 4E). TNBC patients in GSE103091 were divided into two group namely clusterA and cluster (P = 0.04, Fig 4F). Survival analysis between the two groups showed that the OS differences were statistically significant (Fig 4G). Since then, we have demonstrated the stability and accuracy of the above-mentioned key gene screening in different datasets. The expression of hub genes was validated through qRT-PCR and western blot, and the expression of CERCAM, ECM1, HGF, and KLF10 in MDA-MB-231 and BT549 was higher than that in normal breast epithelial cells MCF10A (Fig 5A and 5B). Finally, we analyzed the expression of CERCAM, ECM1, HGF and KLF10 by IHC in TNBC tissues and the results showed that they are positively expressed in TNBC tissues (Fig 5C).

### 3.5 Construction of CAFs Score

To construct the CAFs Score of TCGA-TNBC patients, we used the lasso cox coefficient of 4 hub genes, the formula is as follows:

CAFsScore=0.56750×CERCAM+0.01335×ECM1+0.23449×HGF+0.12938×KLF10

**Fig 6:**
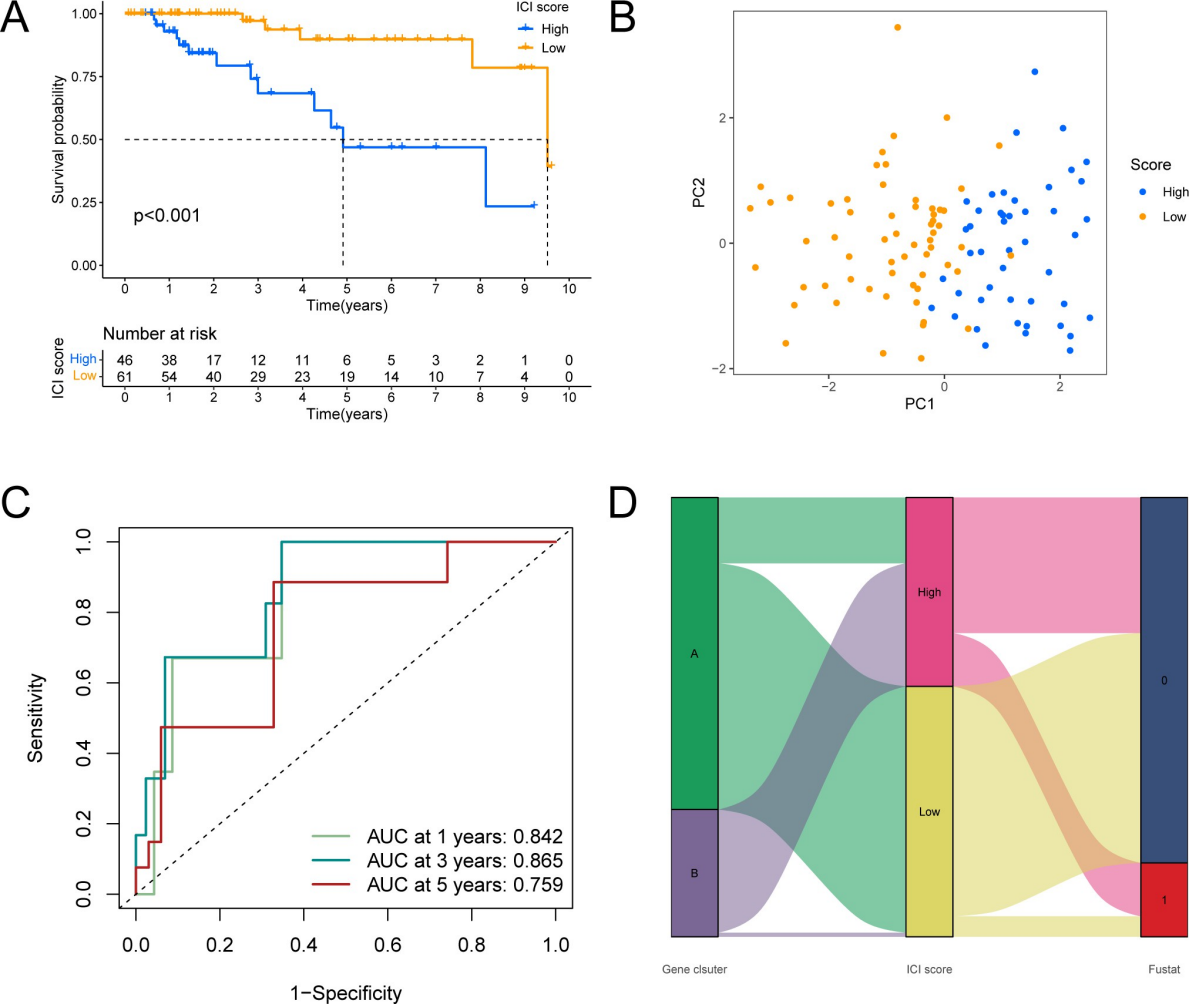
Construction of CAFs Score. (A)Survival of patients in different CAFs Score groups. (B) PCA analysis was used to distinguish the gene distribution of patients in different assessment groups. (C) The area under the ROC curve showed that CAFs had good predictive ability. (D) Sankey plot of survival outcomes in the distribution set of CAFs scores in different subgroups.

Then, according to the surv_cutpoint function, we divided TCGA patients into two groups according to the CAFs Score. The survival analysis showed that the survival of the high score group was significantly worse than that of the low score group (P<0.001, Fig 6A). At this point, we have an accurate score—CAFs Score.

PCA results showed that these four genes could well divide TCGA-TNBC patients into two clusters, and the CAFs Score of the two clusters was significantly different, which was consistent with the results of previous analysis (Fig 6B).The AUC value of CAFs Score in 1.3.5-year survival in TCGA-TNBC patients were 0.842, 0.865 and 0.795, respectively (Fig 6C).As shown in Fig 6D, clusterB was the main source of patients in the high CAFs Score group, clusterA was the main source of patients in the low CAFs Score group, and the prognosis was poor in the high CAFs Score group.

### 3.6 The relationship between CAFs Score and immunity

A total of 39 kinds of immune cells and stromal cells in tumor microenvironment of patients with TCGA-TNBC were evaluated by using R packet “xCELL”, wilson cox was used to examine the differences in the scores of immune cells and stromal cells in patients with high and low CAFS scores. At the same time, the gene expression data of patients’ immune checkpoint were extracted, and the difference of immune checkpoint level between high and low score groups was calculated, P< 0.05 was considered to be statistically significant.

**Fig 7:**
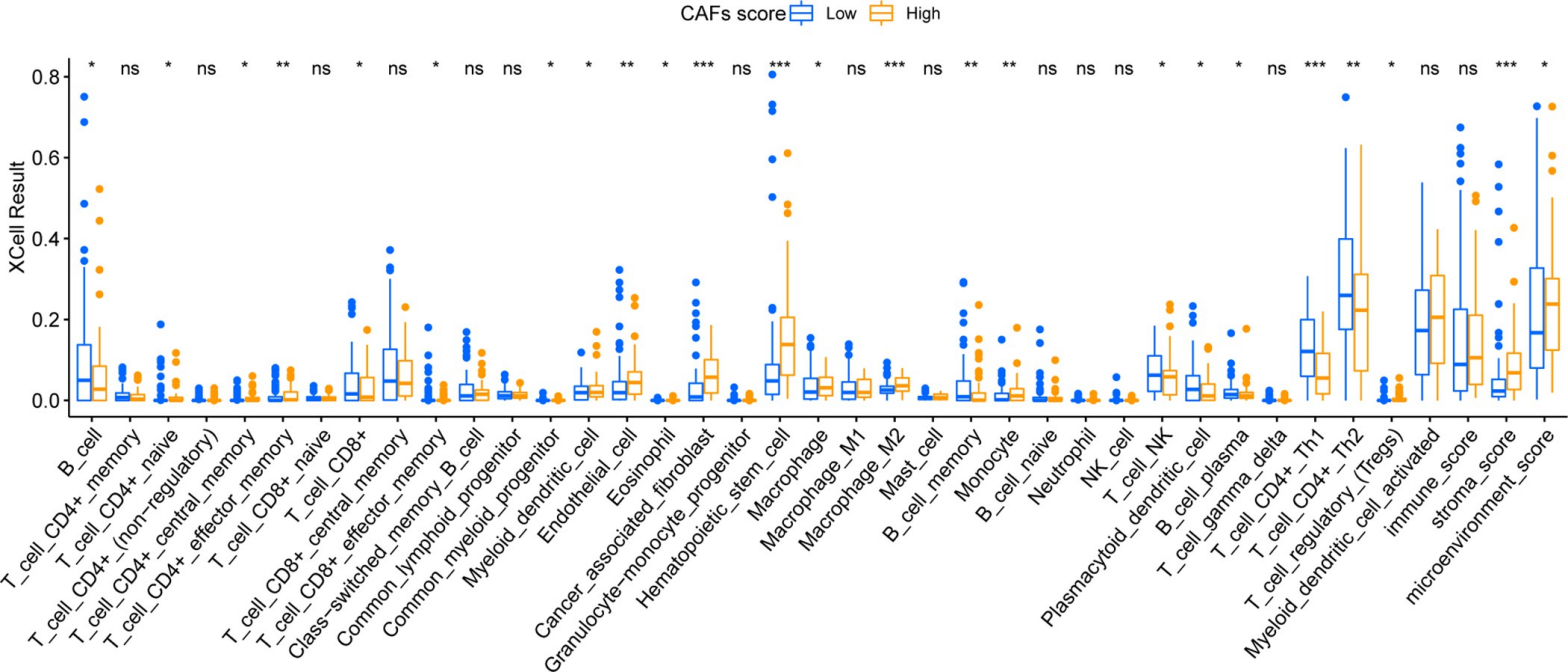
Differences in XCell analysis results among different CAFs Score groups.

**Fig 8:**
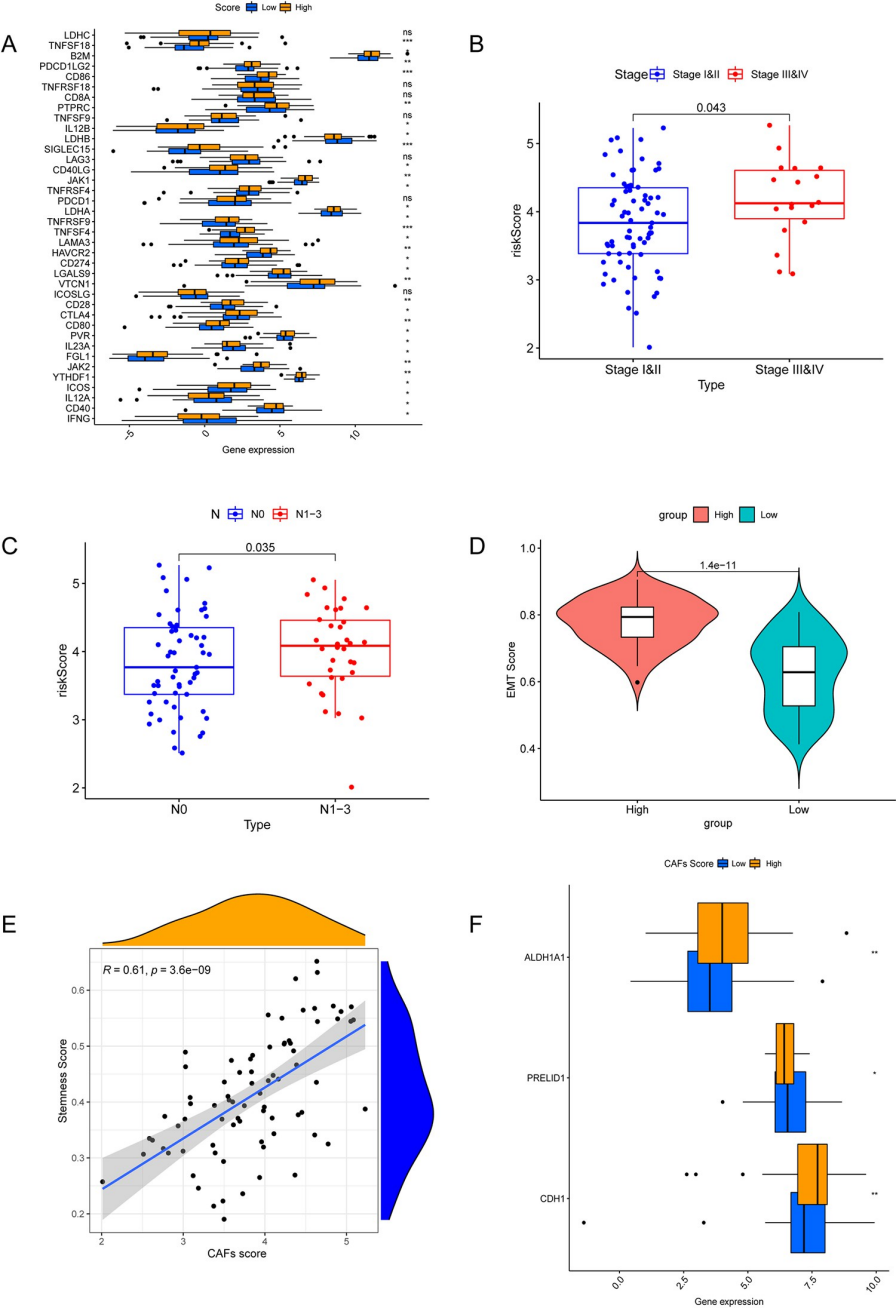
Differences in tumor associated fibroblast characteristics between high and low CAFs Score groups. (A) Difference analysis of immune checkpoint levels between different CAFs score groups. (B, C) Relationship between CAFs score and Stage and N Stage of patients. (D) Relationship between CAFs score and EMT score of TCGA patients. (E) Correlation between CAFs score and patient stemness score. (F) Differential analysis of stem genes among different CAFs groups.

The results show that CAFs can play an immune regulatory role by producing regulatory factors and provide an immunosuppressive environment for tumor cells. To explore the relationship between the CAFs Score constructed in this study and the immune microenvironment, we used xCELL to evaluate 39 kinds of immune cells and stromal cells in patients with TCGA-TNBC, as shown in Fig 7. There were differences in the scores of 24 kinds of immune cells and stromal cells between high and low CAFs groups. The difference in Cancer_associated_fibroblast between the two groups demonstrated the accuracy of our previously constructed CAFS Score. Macrophage_M2, T_cell_CD4+_effector_memory and other important immune cells infiltration were different between high and low CAFS Score groups. At the same time, 31 out of 38 immune checkpoints showed significant difference, and most of them were highly expressed in the high CAFs Score group (Fig 8A). These results suggest that CAFs may affect the immune status of patients with TNBC. The CAFs Score can be used to differentiate TNBC patients with different levels of immune cell infiltration and immune checkpoint expression.

### 3.7 Relationship between CAFs Score and EMT and stemness

The difference of CAFs Score among TNBC patients with different clinicopathologic information was analyzed. Compared with Stage I & II, CAFs Score was higher in TNBC patients with Stage III & IV (P = 0.043, Fig 8B). In N staging, TNBC patients with N1-3 had a higher CAFs Score than TNBC patients with N0 (P = 0.035, Fig 8C). Because the number of TNBC patients in different T and M groups was different, the statistical results were not statistically significant, as shown in S1 and S2 Figs. Based on the annotation gene of EMT related pathway obtained from GSEA database, we obtained the difference of EMT Score between different high and low CAFs Score groups (P = 1.4e-11, Fig 8D). Calculating the correlation between Stemness Score and CAFs Score shows that Stemness Score is positve related to CAFs Score (R = 0.61, P = 3.6e-09, Fig 8E).

### 3.8 The role of CAFs Score in predicting drug sensitivity of tumor therapy

**Fig 9:**
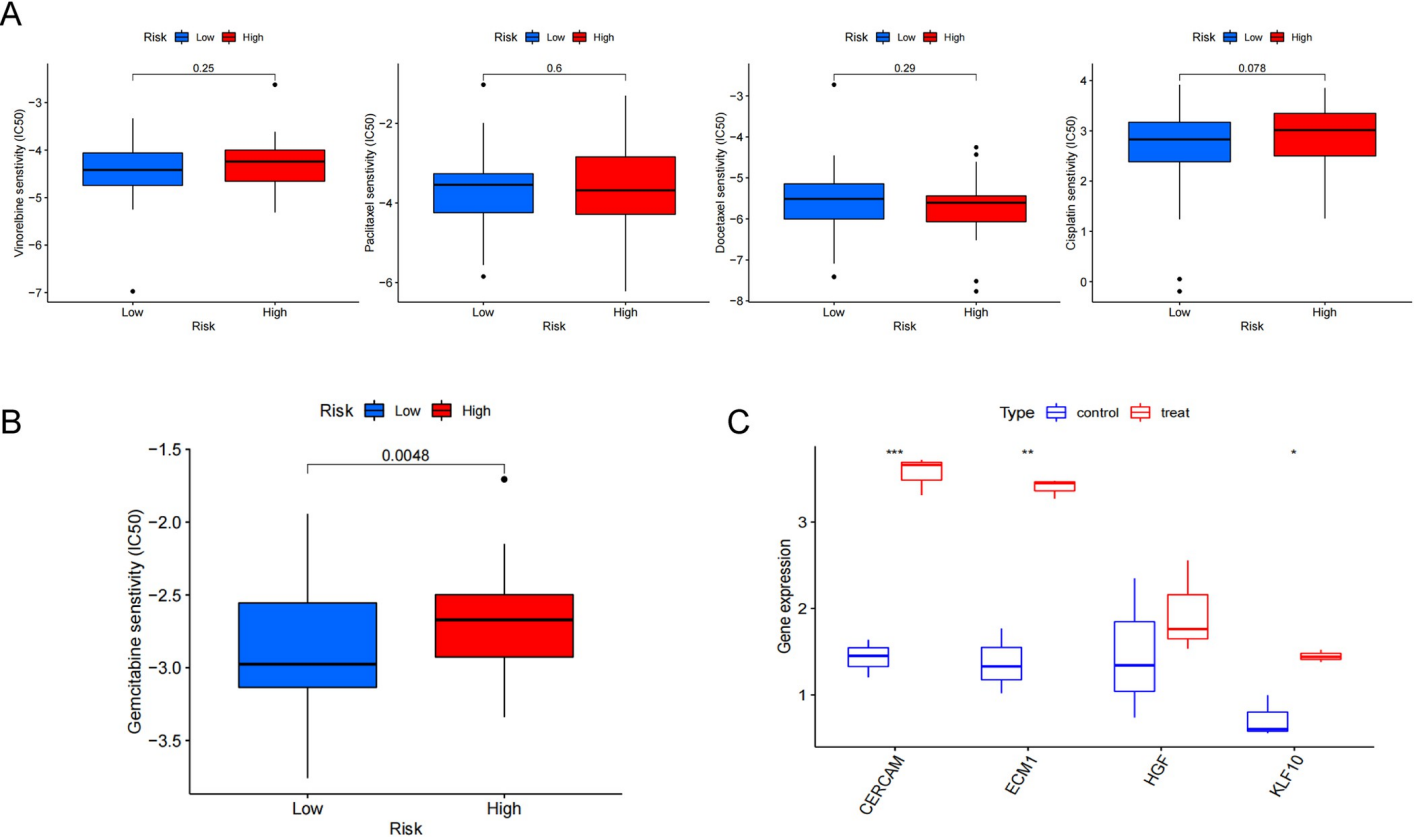
Relationship between CAFs score and chemotherapy drug sensitivity. (A) There was no difference in the sensitivity of patients to Vinorelbine, Paclitaxel, Docetaxel and Cisplatin in the low and high CAFs groups. (B) Patients in the low CAFs score group were more sensitive to Gemcitabine.(C) Expression changes of hub genes before and after treatment with gemcitabine.

pRRophetic was able to analyse the sensitivity (IC50) of patients to 138 chemotherapeutic agents, including those commonly used by TNBC patients. The analysis showed that for many chemotherapeutic agents commonly used in TNBC, such as Vincristine, Paclitaxel, Docetaxel, Cisplatin, etc. (Fig 9A), there was no difference in drug sensitivity between the high and low CAFs Score groups, whereas for Gemcitabine, the low CAFs Score group was more sensitive to Gemcitabine (Fig 9B, P = 0.0048). At the same time, GSE128099 data set was analyzed and in the triple-negative breast cancer cell line supplemented with gemcitabine, four hub genes showed higher expression than previously, among which CERCAM (P = 0.00029), ECM1 (P = 0.0072) and KLF10 (P = 0.027) were statistically significant, while HGF showed an increasing trend but no statistical significance (Fig 9C). Consistent with our previous work, the upregulation of these genes may suggest a reduction in the sensitivity of cancer cells to gemcitabine following treatment. We suggest that it is possible that these four CAFs-related genes are involved in regulating Gemcitabine resistance.

## 4 Discussion

Patients with TNBC cannot benefit from endocrine therapy, targeted therapy and other conventional treatments for breast cancer, and in recent years, researchers have been trying to explore other effective treatments and targets for TNBC. As the most important mesenchymal cells in the tumor microenvironment, tumor-associated fibroblasts have multiple pro-cancer mechanisms [14],we therefore believe it is of great interest to develop a study from this perspective. Based on scRNA sequencing, the TCGA-TNBC database and the GEO database, a score related to CAFs in TNBC patients was constructed. To some extent, the CAFs Score quantifies the immune microenvironment, EMT stemness and sensitivity to relevant therapeutic agents in TNBC patients. The results of the study suggest that the CAFs Score can accurately predict the prognosis of TNBC patients and may guide the selection of therapeutic agents for TNBC.

Firstly, we analysed the scRNA sequencing dataset GSE118389, clustered and annotated each cell in the dataset to identify CAFs-associated clusters, analysed the marker genes of CAFs-associated clusters, combined with the TNBC data in the TCGA database for cox regression analysis, lasso analysis, etc. And finally, we obtained the four most stable prognostic genes associated with CAFs in TNBC: CERCAM, KLF10, ECM1,HGF.

CERCAM, also known as cerebral endothelial cell adhesion molecule, is a cell adhesion molecule, and studies have shown that CAFs enhance epidermal cell adhesion and promote tumor cell metastasis [15, 16]. CERCAM is upregulated in a variety of malignant cells and in TME [17], and has been defined in some cancers as a prognostic marker [18]. In HNSCC, CERCAM promotes malignant biological behavior of tumor and M2 polarized immune infiltration of macrophages, which are independent risk factors for predicting OS in patients [19].However, CERCAM has not been shown to be an independent prognostic risk factor in breast cancer.

KLF10, also known as TGFβ inducible early gene-1 (TIEG), is an oncogene that acts as a gene transcription regulator. Its classical pathway of action activates the TGFβ-Smad signaling pathway mainly through inhibition of Smad7 expression and activation of Smad2 expression and activity [20], and studies in breast cancer have shown that its expression level is negatively correlated with breast cancer stage [21].Wei et al. also concluded that KLF10 inhibits EGFR transcription and the EGFR pathway to suppress breast cancer invasion and metastasis [22].Similar to this study, the researchers established a gene prediction model for CAFs in lung squamous cell carcinoma(LUSC). KLF10, as one of the key genes, can predict the prognosis and treatment response of patients with LUSC [23].

The full name of ECM1 is extracellular matrix protein 1, and studies have shown that this gene is overexpressed in many malignancies of epithelial origin, including colorectal, thyroid, and hepatocellular carcinomas [24, 25], and also breast cancer [26, 27].Overexpression of ECM1 can promote the stemness phenotype of tumor cells as well as the EMT process [28]. It has also been shown that in breast cancer, ECM1 can regulate the actin cytoskeleton structure of invasive breast cancer cells through the regulation of S100A4 and Rhoa [29]. A total of four subtypes of breast cancer-associated fibroblasts (BCAFs) have been identified in the current study [30], The distribution of each subtype and the expression of related factors are related to the stromal histological features and molecular subtypes of breast cancer, with CAF-S1 and CAF-S4 subtypes being prevalent in triple-negative breast cancers. Among them, the CAF-S4 subgroup is mainly involved in regulating processes such as the actin backbone and muscle contraction [31]. We speculate that ECM1 may play a role in the regulation of biological behaviour of breast cancer cells by CAF-S4 subpopulation cells, but the exact mechanism still needs to be explored.

HGF is a growth factor that can be produced by tumor stromal cells and plays an important role in promoting malignant tumor progression and metastasis [32, 33], with the HGF/c-Met signaling pathway being the most classical pathway in which it functions [34–36], and the specific binding of HGF to c-Met triggers a series of responses that promote cell migration, induce angiogenesis and promote stromal degradation and invasiveness. HGF/c-Met is already a very promising target for cancer therapy. Currently, Boswellia frereana, a drug, has been reported to target the HGF/c-Met signaling pathway to inhibit the biological behavior of TNBC cells such as migration, adhesion and angiogenesis [37].Recent studies have shown that radiation can activate the HGF/C-met signaling pathway, and that high expression of HGF and c-Met is associated with worse recurrence-free survival in breast cancer patients receiving radiotherapy [38]. BCAFs can produce a variety of MMPs that induce invasion, migration and metastasis of breast cancer cells [39], and HGF antagonists have been shown to downregulate MMP-9 activity in lung cancer cells [40], leading us to speculate that HGF may play an important role in the secretion of MMPs by BCAFs to directly or indirectly induce invasion and metastasis of breast cancer.

In addition to bioinformatics analysis and the current status of gene research, we use PCR and WB experiments found that these 4 genes are highly expressed in TNBC cell lines. Furthermore, IHC experiments showed that these 4 genes are positively expressed in TNBC tissues. At this point, we believe that it is feasible to further establish CAFs-related scores. In this study, the CAFs Score was constructed using the above four genes in the hope of grouping TNBC patients by BCAFs and exploring the differences in tumor tissues of triple negative breast cancer patients with different CAFs scores to find a breakthrough in treatment. As mentioned in the previous discussion, CAF-S1 and CAF-S4 subgroups are prevalent in TNBC, and the CAF-S1 subgroup is mainly involved in regulating genes related to cell adhesion and extracellular matrix as well as immune response, promoting the formation of an immunosuppressive environment [30, 31]. Recently, a team in our country has found that biglycan (BGN), an extracellular protein, is a specific factor secreted by CAFS and a prognostic marker and immunotherapy target of triple-negative breast cancer [41]. Further, in pan-cancer analysis, BGN was also an unfavorable predictor of overall survival and response to immunotherapy in patients with multiple malignancies besides triple-negative breast cancer [42]. It has also been shown that some BCAFs lacking Tiam1 protein expression produce high levels of osteopontin (OPN), which in turn promotes epithelial-mesenchymal transition, tumor stem cell phenotype [43]. We hypothesized that TNBC patients with different CAFs Scores may have different immune status, EMT status and stemness intensity. The results obtained from the analysis were as we hypothesized, important immune cell infiltrations including Macrophage_M2, T_cell_CD4+_effector_memory differed between high and low CAFs Score groups, 31 out of 38 immune checkpoints differed between score groups and most were highly expressed in the high CAFs Score group. Analysing the differences in these states between the high and low scoring groups not only demonstrates that CAFs can indeed influence the immune status of TNBC patients, but also the accuracy of the score constructed in this study and its ability to differentiate TNBC patients with different immune status, providing some reference for precise treatment. Based on the results of the analysis, we venture to speculate that the application of different immunosuppressive agents in combination with CAFs-related inhibitors for TNBC patients with different CAFs score levels may increase the response of TNBC patients to immunotherapy and thus improve the prognosis of TNBC patients.

We then analyzed the relationship between CAFs Score and different clinicopathological profiles, as well as the correlation between their EMT scores and tumor stemness characteristics scores, both of which were obtained. The CAFs Score was higher in TNBC patients at higher stage and higher N-stage, and the EMT as well as the stem cell characteristics score were higher in the high CAFs Score group than in the low CAFs Score group. Further arguing our speculation. The CAFs Score obtained in this study can differentiate TNBC patients with different immune status, different clinical stages, different EMT and stemness status to guide precise treatment.

Studies have shown that differences in sensitivity to chemotherapeutic agents depend not only on the cancer cells themselves but also on the tumor microenvironment [44–46]. CAFs can promote resistance of breast cancer cells to a variety of chemotherapeutic agents, endocrine therapeutic agents [47, 48]. CAFs also have multiple pathways to promote drug resistance [49, 50]. In this study we analyzed the difference in sensitivity to chemotherapy between the high and low CAFs Score groups, and we found no difference in the sensitivity of these two groups to commonly used chemotherapeutic agents for TNBC such as Vincristine, Paclitaxel, Docetaxel and Cisplatin, while the low CAFs Score group was more sensitive to Gemcitabine. We speculate that these four CAFs-related genes are likely to be involved in regulating Gemcitabine resistance. The main chemotherapy regimens currently used for the relief of advanced TNBC include Paclitaxel combined with Capecitabine, Gemcitabine combined with Platinum, Paclitaxel combined with Platinum, Vincristine alone, Gemcitabine alone, etc. Given that the high CAFs Score group is less drug-sensitive to Gemcitabine and more likely to develop resistance, analysis of the GSE128099 data showed increased expression of these four genes in triple-negative breast cancer cells treated with Gemcitabine. we believe that Gemcitabine-containing chemotherapy regimens should be avoided in this group of patients with TNBC. This could guide the choice of chemotherapeutic agents for advanced TNBC, which we believe is of great interest.

In summary, we identified a set of triple-negative breast cancer cells associated with tumor-associated fibroblasts by single-cell sequencing dataset, and screened four hub gene associated with CAFs in TNBC based on marker genes of these cells in combination with TCGA database. Based on these four genes, we constructed CAFs Scores and showed differences in prognosis, immune status, EMT status and stemness scores between the high and low CAFs Score groups, and we hypothesized that these four CAF-related genes may be associated with gemcitabine resistance in TNBC patients and that patients in the high CAFs Score group should avoid Gemcitabine-containing chemotherapy. This paper is primarily an exploration based on public databases. This paper is mainly based on the exploration of public databases, which still has some limitations, although the experimental verification of gene expression, but the lack of mechanism-related analysis and exploration. It is hoped that the results of the analysis based on big data can provide help for the exploration of CAFs in TNBC.

## 5 Conclusion

In this study, we identified a group of TNBC cells associated with CAFs through analysis of scRNA-seq data, and screened four CAFs-related hub genes in TNBC based on the marker gene of this group of cells and bulk data. The follow-up analysis suggested that our CAFs Score correlated with the prognosis ofTNBC patients, associated immune levels, stemness scores and EMT levels, and that these four CAFs-related genes may be involved in regulating Gemcitabine resistance in TNBC patients.

## Supporting information

S1 Fig The number of TNBC patients in different T groups. (PDF)

S2 Fig The number of TNBC patients in different M groups. (PDF)

S1 Table Information of the hub genes of the different clusters. (XLSX)

S2 Table Information on differentially expressed genes in clusterA and clusterB. (XLSX)

## Abbreviations

GEO: Gene Expression Omnibus
TNBC: Triple negative breast cancer
TME: Tumor microenvironment
TCGA: The Cancer Genome Atlas
LAR: luminal androgen receptor
ECM: extracellular matrix
CAFs: tumor-associated fibroblasts
EMT: epithelial-mesenchymal transformation
scRNA-seq: Single cell RNA sequencing data
IHC: immunohistochemical staining
CDF: cumulative distribution function
OS: overall survival
