# Supplementary figures and images for: Establishment of a tumor-associated fibroblast associated gene score based on scRNA-seq to predict prognosis in patients with triple-negative breast cancer
Source: PLoS One. 2024 Oct 17;19(10):e0311801. doi: 10.1371/journal.pone.0311801 (PMC11486389; doi:10.1371/journal.pone.0311801)

Type 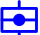 T1-2 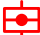 T3-4

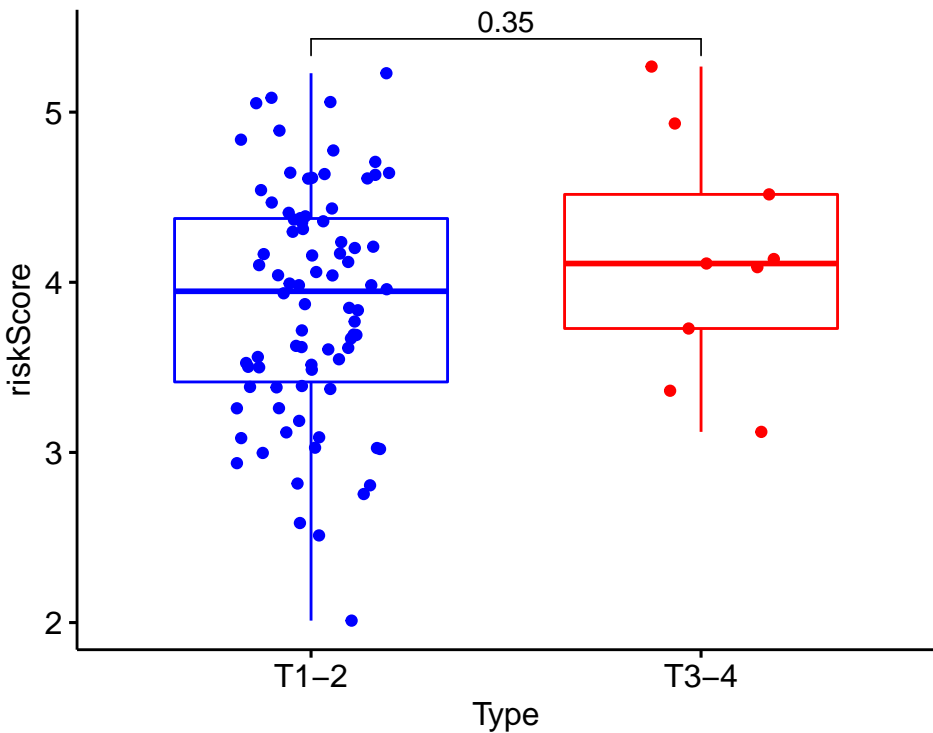

Supplement: S1 Fig — (PDF) [file pone.0311801.s001.pdf]

Type M0 M1

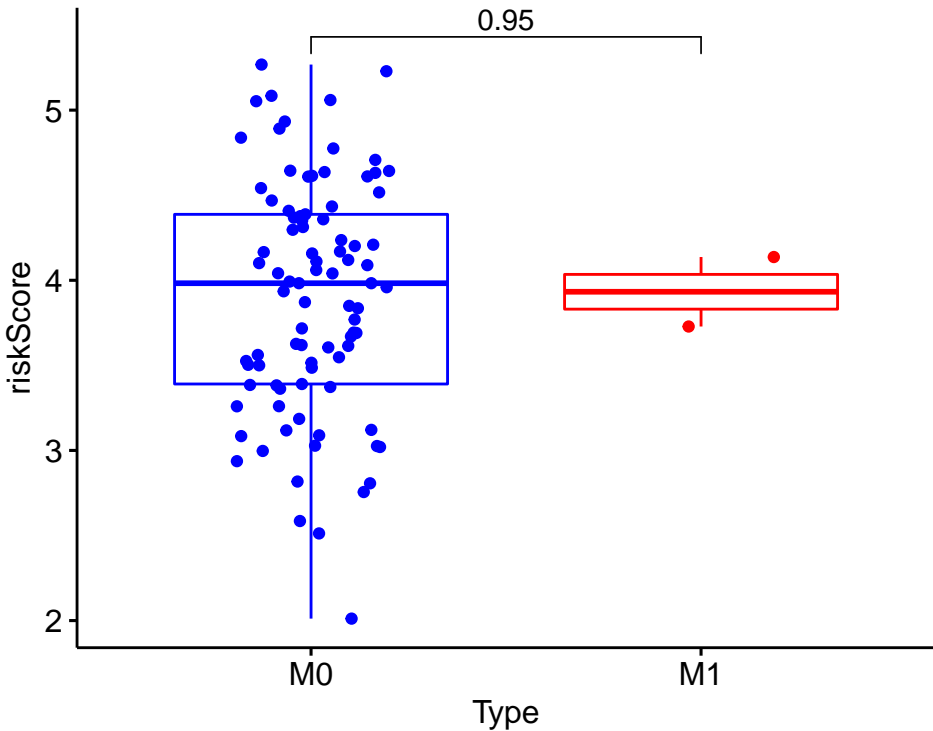

Supplement: S2 Fig — (PDF) [file pone.0311801.s002.pdf]
